# Evaluation of Pharmacy Resident Burnout Based on Weekend Residency Staffing Requirements: A Pilot Study

**DOI:** 10.3390/pharmacy13060153

**Published:** 2025-10-27

**Authors:** Andrew C. Hean, Jamie Kneebusch, Casey Tiefenthaler, Kelly C. Lee

**Affiliations:** 1 College of Pharmacy, Western University of Health Sciences, 309 E 2nd St, Pomona, CA 91766, USA; ahean@westernu.edu; 2 Skaggs School of Pharmacy and Pharmaceutical Sciences, University of California San Diego, 9255 Pharmacy Lane, La Jolla, San Diego, CA 92093, USA; jkneebusch@health.ucsd.edu; 3 Department of Clinical Pharmacy, University of California, San Diego Health, 200 W Arbor Drive, San Diego, CA 92103, USA; cmtiefenthaler@health.ucsd.edu

**Keywords:** burnout, residency, resident, pharmacy, pharmacist, staffing, workload, resilience, wellness

## Abstract

Research surrounding impacts of staffing on pharmacy residents is limited. This prospective survey study aims to elucidate relationships between burnout and weekends staffed among California pharmacy residents. Postgraduate year 1 and 2 (PGY1 and PGY2) pharmacy residents completed electronic surveys in August 2023 and February 2024. The primary outcome was the difference in burnout score changes based on weekends required to staff (measured using the Maslach Burnout Inventory-Human Services Survey for Medical Personnel). Secondary subgroup analyses measured differences in burnout scores by the overall cohort, no weekend staffing vs. weekend staffing required, PGY1 vs. PGY2, and by changes in planned professional pursuits. Of 66 respondents, no significant differences in burnout scores were observed based on the number of weekends required to staff. Final mean emotional exhaustion (EE), but not depersonalization (DP) or personal accomplishment (PA), scores were significantly higher for all residents combined, increasing from 24.8 (SD 10.2) to 28.4 (SD 11.5). Final mean EE scores were also significantly higher among PGY2s compared to PGY1s, at 35.1 (SD 0.70) vs. 25.8 (SD 12.0), respectively. Final mean burnout scores were significantly worse in those becoming less likely to pursue board specialty certification across all domains, with EE = 32.6 (SD 6.50), DP = 4.29 (4.79), and PA = 36.3 (SD 3.21). Based on these results, staffing intensity alone may not be associated with burnout among California pharmacy residents, but PGY2 pharmacy residents may be at higher risk of burnout. Higher burnout scores may predict the likelihood of pursuing board specialty certification. Future studies assessing additional confounding factors with a broader scope are needed to fully define risk factors for burnout in pharmacy residents.

## 1. Introduction

Pharmacy residents in the United States (U.S.) face a unique set of stressors during their training, including direct patient care, longitudinal research and quality improvement projects, and didactic and experiential teaching for trainees and other healthcare professionals. In the U.S., graduates of an accredited pharmacy degree program or those with a foreign graduate equivalent certificate may choose to pursue additional postgraduate training, commonly through American Society of Health-System Pharmacists (ASHP)-accredited pharmacy residency programs [1]. Per ASHP definition, these 52-week training programs aim to “provide hands-on decision making and experience working with other healthcare professionals under the supervision of an experienced preceptor. [Pharmacy residency] also includes focused mentoring, leadership development, research experience, and prepares [residents] for more advanced pharmacy positions [2].” Those who complete a general postgraduate year 1 (PGY1) pharmacy residency may pursue an additional 52-week postgraduate year 2 (PGY2) pharmacy residency focused on a specific specialty area [1]. However, aside from mandated ASHP requirements and learning objectives, programs may still vary widely in implementation. For example, one common requirement in many U.S. pharmacy residency programs is “staffing” where residents may independently work pharmacist shifts. Staffing may occur on weekends, which can cause pharmacy residents to exceed a standard 40 h workweek in direct patient care hours alone. Although examples of how staffing can factor into residency programs’ requirements and maximum hours worked by residents are mentioned in ASHP accreditation guidance [1], it is not a required component of residency programs. Therefore, workload requirements may vary among programs based on variations in staffing hour requirements alone.

Survey-based studies of pharmacy residents have found varying results in regard to the impact of hours worked on wellbeing in the past decades [3,4]. Pharmacy residents working over 60 h per week have also exhibited high levels of perceived stress, depression, hostility, and dysphoria [5]. Implementation of work-hour limits in physician residencies is associated with improved physician safety and health [6,7]. However, no such large studies exist for pharmacy residents, despite the implementation of duty hour limits of 80 h per week by the American Society of Health-Systems Pharmacists (ASHP) [1]. Some have proposed these limits are inappropriately adapted from United States physician resident duty hour standards given pharmacy and physician residents’ differences in responsibilities and focus [8].

Prior research has elucidated the relationship between increased work demands and resulting burnout in the context of the Job Demands–Resources (JD-R) model. In essence, the JD-R model defines occupations as having either demands and resources: inadequate job resources will inadequately buffer job demands, leading to occupational strain such as burnout [9]. Burnout is defined in the World Health Organization’s International Classification of Diseases, 11th Edition, as an occupational phenomenon “resulting from chronic workplace stress that has not been successfully managed” characterized by three dimensions: “feelings of energy depletion or exhaustion,” “increased mental distance from one’s job, or feelings of negativism or cynicism related to one’s job,” and “reduced professional efficacy.” [10]. Under the JD-R model, burnout leads to negative emotional and physical health outcomes on an individual level but also may be deleterious for organizations [11]. In the healthcare setting, these impacts may be concerning for both patient and professional wellbeing.

Notably, excessive work hours have been associated with burnout in pharmacists [12], drawing concerns for burnout in pharmacy residents. A single-center study found that burnout rates among pharmacy residents were as high as 74% which was associated with falling asleep at work and neglecting institutional guidelines [13]. Pharmacy resident burnout is also associated with higher cynicism scores [14]. These findings are especially concerning considering pharmacy residents may exhibit higher rates of depression and suicidal thoughts compared to the general population [15].

Recent survey data shows concerning trends of pharmacy residents dropping out of PGY1 residency in addition to declines in matching for PGY2 residencies: mental health, workload, and work environment/culture are cited as potential work-related contributors [16]. In particular, burnout has been endorsed by some residency program directors (RPDs) as key drivers of these issues in resident retention [16]. Critical evaluation of residency components, such as weekend staffing, may help guide residency program directors in preventing burnout and preventing further declines in the pursuit of residency training. However, alleviating the impact of work stress to reduce the burnout of pharmacy residents may be challenging, as there is concern that the quality of pharmacy residency training could be reduced [17]. The current gap in the literature describing which residency components are especially associated with burnout should be addressed. In particular, given variations in weekend staffing program requirements, studies are needed to determine whether weekend staffing workload is associated with increased burnout in pharmacy residents. The objective of the study is to examine whether pharmacy residents in the state of California exhibit different levels of burnout based on their weekend staffing requirements.

## 2. Materials and Methods

This was a prospective, longitudinal survey study of the 2023–2024 cycle of pharmacy residents enrolled in any ASHP-accredited residency program in California. Residency Program Directors (RPDs) were e-mailed and requested to send the baseline survey to their residents with an additional reminder to RPDs one week prior to survey closing. Participants completed a survey administered at two timepoints during the 2023 to 2024 residency cycle corresponding to beginning and halfway points of the residency year: August 2023 (baseline) and February 2024 (final). Surveys were open for approximately 3 weeks for respondents to complete. Participants who completed the baseline survey were contacted directly by e-mail to participate in the final survey with a unique survey link. A reminder was also sent one week prior to its closing. Regardless of consent to participate, all respondents reporting their e-mail (used only for this purpose and to remove duplicate entries) were included in drawings for electronic gift cards following each survey. Fifteen $25 gift cards were raffled for the baseline survey and fifteen $50 gift cards were raffled for the final survey. The surveys were administered using Qualtrics^XM^ [18]. The Institutional Review Board (IRB) at the university deemed this study exempt from IRB approval.

A contact list for Residency Program Directors (RPDs) was compiled in July 2023 using the online ASHP Residency Directory and was supplemented by manual searches on program websites as needed [19]. A total of 200 unique RPD contacts representing 221 possible programs resulted from this list. Exclusion criteria was non-completion of all 22 questions of the Maslach Burnout Inventory Human Services Survey for Medical Personnel (MBI-HSS [MP]) on either survey. Additional attempts to contact any e-mails initially returning in error were not made as to standardize outreach methods.

The 41-item baseline survey gathered responses on demographics, residency program characteristics, staffing model, burnout as measured by MBI-HSS [MP], Likert scale questions regarding career satisfaction (1 = Strongly disagree, 2 = Somewhat disagree, 3 = Neither agree nor disagree, 4 = Somewhat agree, 5 = Strongly agree), and likelihood of further professional pursuits through either Board of Pharmacy Specialty (BPS) certification or further postgraduate training. For respondents who were unable to specify the exact number of weekends they are required to staff (i.e., their staffing model is based on frequency of weekends), the expected number of weekends staffed total was calculated by the investigators based on the frequency reported out of a 52-week long residency year. Any specific details were factored in depending on additional free-text comments from respondents (e.g., the first 4 weeks were not accounted for if respondents reported not working weekends for the first month). Free-text response handling for all questions was reviewed by two investigators for agreement. To preserve respondents’ anonymity, PGY2s were not asked to specify their specialty area since some PGY2 specialties exist in only one or limited programs within California. The Likert Scale career satisfaction question responses are not reported as they were exploratory and not used for this analysis. The baseline survey, excluding the copyrighted MBI-HSS [MP] items, is available in Appendix A.

The MBI-HSS [MP] is a 22-question-item survey tool that has been validated in numerous worker groups including healthcare professionals [20]. The MBI-HSS [MP] survey consists of three distinct domains: Emotional Exhaustion (EE), Depersonalization (DP), and Personal Accomplishment (PA). A higher score in the EE and DP domains and lower score in the PA domain are indicative of higher degrees of burnout. Total scores of each domain were calculated and used to determine differences between staffing model cohorts. As to protect anonymity of respondents from programs with easily identifiable staffing or compensation day models, the specific free-text responses and their recategorization are not reported here.

The final survey was identical to the baseline survey except without demographic questions. In the case of multiple entries identified by duplicate e-mails, only responses completing the MBI-HSS [MP] were kept and the latest entry within the survey window was kept. Survey data were only accessible by two investigators using password-protected web logins. The survey tool was beta-tested by three individuals.

The primary outcome was the difference in MBI-HSS [MP] score changes from baseline to final survey, based on the numbers of weekends required to staff. Secondary outcomes included the change in mean MBI-HSS [MP] scores of the following subgroups: (1) the overall combined cohort, (2) respondents reporting no weekend staffing vs. any required weekend staffing, and (3) PGY1s vs. PGY2s at both timepoints (if required to participate in weekend staffing). Lastly, the difference in final survey MBI-HSS [MP] scores, based on change in professional pursuit plans between the two surveys, was also analyzed. The categorization of change in professional pursuits was defined based on how answers changed (or remained unchanged) between the baseline and final surveys between “Yes”, “No”, or “Maybe.” In particular, this change was classified on a spectrum: a change from “yes” to “unsure” or “no” was considered less likely; a change from “no” to “unsure” or “yes” were considered more likely; a change from “unsure” to “yes” was considered more likely whereas “unsure” to “no” was considered less likely. Unchanged responses between the two survey timepoints were categorized as unchanged.

Professional pursuits included questions regarding plans to pursue Board of Pharmacy Specialty certification or additional postgraduate training. 

Descriptive statistics were used for demographic characteristics (summarized by %, mean, and standard deviation). A one-way between-groups analysis of covariance (ANCOVA) was conducted to compare the change in burnout scores between baseline and final survey administrations for the primary outcome. The independent variable was the number of weekend staffing shifts, and the dependent variable was the respondents’ score at the final administration. Respondents’ scores at baseline were used as the covariate. Changes in MBI-HSS [MP] scores in the overall cohort as a secondary outcome was analyzed by paired t-test. Two-sided t-tests were used to compare: (1) no weekend staffing vs. any weekend staffing and (2) PGY1 vs. PGY2 scores at each survey timepoint. One-way analysis of variance (ANOVA) was used for differences in MBI-HSS [MP] scores by change in professional pursuit plans. All statistical analyses were conducted using IBM SPSS Version 28 (Armonk, NY, USA) [21].

## 3. Results

### 3.1. Study Participation

During outreach to RPDs, 14 emails were returned as invalid and one RPD opted out based on their residents not staffing weekends. Among all programs, there was a total of 502 available advertised resident positions. The MBI-HSS [MP] portion of the baseline survey was completed by 107 respondents. Of these, 66 of these respondents (13.1% of the potential 502 resident positions advertised) completed the MBI-HSS [MP] portion of the final survey and were included in the final analysis.

### 3.2. Baseline Characteristics

Of the 66 respondents who completed the MBI-HSS [MP] in both surveys, 11 (16.7%) self-identified as having no weekend staffing requirement while 55 (83.3%) reported a weekend staffing requirement. Full demographic information separated by these staffing requirements is displayed in Table 1.

### 3.3. Primary Outcome

After adjusting for baseline scores, there was no significant difference in EE based on number of weekend staffing shifts, F (18, 46) = 1.37, *p* = 0.19, partial eta squared = 0.35. There was a weak relationship between the baseline and second administration on EE, as indicated by a partial eta squared value of 0.134. Due to violation of homogeneity of variance tests, ANCOVA analyses were not conducted to detect differences in DE or PA scores.

### 3.4. Secondary Outcomes

#### 3.4.1. Overall Cohort Changes by Paired *t*-Test

When compared by paired *t*-test, there were significant differences in EE scores between the baseline survey (24.8, SD 10.2) and final survey (28.4, SD 11.5) (*p* = 0.036). However, mean DP and PA scores were not statistically significantly different between the two timepoints (Table 2).

#### 3.4.2. No Weekend Staffing vs. Any Required Weekend Staffing

Between respondents with no weekend staffing vs. any required weekend staffing, there were no significant differences found at either survey timepoint (Table 2).

#### 3.4.3. PGY1 vs. PGY2

Among the PGY1 and PGY2 respondent cohorts required to staff weekends at the baseline survey, mean MBI-HSS [MP] scores in all domains appeared similar between the two residency year groups (Table 2). However, significant differences were observed for EE scores at the final survey timepoint, with a PGY1 mean score of 25.8 (SD 12.0) and PGY2 mean score of 35.1 (SD 0.70) (*p* = 0.007) (Table 2).

#### 3.4.4. Stratification by Changes in Planned Professional Pursuits

There were significant differences in the final survey mean scores for all three domains of the MBI-HSS [MP] when stratified by change in BPS certification plans (Table 3). Respondents becoming less likely to pursue BPS certification had the highest final timepoint burnout scores across domains (higher EE and DE and lower PA) compared to those more likely to pursue or with unchanged plans to pursue. However, differences were not statistically significant when this analysis was completed based on likelihood of further postgraduate training (Table 3). Notably, the majority of the respondents had unchanged plans to pursue either of these professional pursuits (Table 3).

## 4. Discussion

To our knowledge, this pilot study is the first to examine the relationship between weekend staffing and burnout scores among pharmacy residents in a longitudinal manner. Previous studies have assessed the impact of work hours or overnight on-call hours on pharmacy residents, but did not directly measure burnout [5,22,23]. Future studies may benefit from examining residency components in a more qualitative manner rather than quantitative. Staffing experiences may vary widely depending on the type of activities and level of independence during staffing that may impact burnout levels, which may explain the lack of observable differences in MBI-HSS [MP] scores based on the number of weekends required to be staffed.

Secondary analyses revealing differences in EE aligns with the prior literature. One survey study of health system pharmacists noted the highest percentage of domains among pharmacists that fell into a threshold for high burnout was EE, followed by PA, and then DP [24]. Similar results were reported in a survey of Canadian hospital pharmacists but with PA and DP having similar percentages [25]. A single-center study of pharmacy residents within two months of the end of residency found nearly three-fourths of the 58 residents in the cohort had mean EE or DP scores that met the commonly used cutoff in the prior literature for burnout, denoted by an EE score of 27 or more and DP score of ten or more [13]. Our results for the entire combined cohort met these cutoffs at the final survey timepoint for EE but not DP or PA, suggesting that the latter part of the residency year may be the optimal timepoint to implement anti-burnout initiatives focused on EE.

In the context of JD-R theory, work engagement is a motivational process in contrast to burnout that leads to favorable organizational outcomes [11]. Considering EE scores are commonly of focus in the literature among pharmacy residents, future studies should consider examining the relationship between increasing PA scores (or other work engagement measures) and alleviation of burnout in pharmacy residents. As some studies have demonstrated a curvilinear relationship where too little work demands (“underload”) can negatively impact job satisfaction [26], the absence of common residency components like weekend staffing should also be investigated as a potential contributor to burnout or job dissatisfaction among pharmacy residents.

Unlike many prior burnout studies [27], we did not define thresholds for scoring given the latest edition of the Maslach Burnout Inventory advises no longer using cutoffs to classify categories of burnout [28]. The measurement of burnout as a binary phenomenon as opposed to a spectrum has been debated, as dichotomization may inaccurately characterize the effects of burnout (i.e., it may be inappropriate to use a score to state someone is or is not burned out) [29,30]. The survey response rate in this study was adequate for a predicted 45 respondents needed to detect a 5.0 point change in the EE domain of the MBI-HSS [MP], assuming a SD of 10.0 on each survey timepoint. However, due to the lack of prior literature to inform a meaningful expected change in burnout scores in this population, the observational nature of this study, and the finite number of California pharmacy residents in this residency cycle that can be recruited, the applicability of the statistical significance based on the predicted power of this pilot study is limited. Future studies should consider validating the magnitude of changes in burnout scores among pharmacy residents that were observed in this study.

In a single-center cross-sectional study of pharmacy residents, there was no difference in frequency of plans to obtain advanced certification or further postgraduate training based on categories of burnout [13]. Our longitudinal study results were mixed in terms of the relationship between burnout and postgraduate plans for residency. While baseline MBI-HSS [MP] scores showed no notable differences, the final survey showed the highest degree of burnout in all MBI-HSS [MP] domains in those who self-reported ambivalence in their plans to pursue BPS Certification. However, our study and the currently available literature do not provide clear explanation on whether burnout itself is a causative determinant of plans for professional pursuits among pharmacy residents, or if both of these variables are mostly dependent on other factors. For example, in the context of JD-R theory, it is possible that higher burnout scores are a reflection of the presence of other job demands unbalanced by job resources, and this lack of resources could discourage post-residency endeavors through a variety of avenues amongst early career trainees. Future studies would benefit from assessing pharmacy residents’ professional pursuits in this manner and utilizing such occupational frameworks. Of note, one limitation in the categorizations used in our study is the lack of differentiation between those who had unchanged plans to either pursue or not pursue these professional pursuits. Rather, our recategorization focuses on residents whose plans changed or did not change. Interestingly, one prior nationwide cross-sectional survey of the 2017–2018 cycle of PGY1s demonstrated lower burnout scores in those pursuing or planning to pursue additional postgraduate training compared to those who were not [31]. If burnout itself is a causative factor for lack of professional engagement in residents, burnout prevention could be an avenue for future implementation studies to explore in encouraging additional postgraduate professional pursuits or preventing changes in individuals who are already borderline in these decisions. These types of initiatives may especially be pertinent due to recent data demonstrating decreasing PGY2 matches [16].

We hypothesize that the higher EE scores among PGY2s compared to PGY1s at the final survey timepoint may be related to pressures of securing a job post-specialization and total time spent in residency. The existing literature following pharmacy residents after residency is generally focused on career after residency. A national United States survey of 473 RPDs found the most important characteristic of a successful resident was interpreted as obtaining a clinical position after residency [32]. Notably, another study examined associations of successful residency project publication with post-residency career type and found links between publication and career type in PGY2 pharmacy residents but not PGY1 pharmacy residents [33]. This may be an area of interest to study for additional burnout factors in PGY2s, given our EE findings among PGY2s at the midpoint of residency year.

As a survey-based analysis, this study also has limitations related to self-reporting, selection bias, and confounding factors. Although the study spans programs statewide and may capture a wide variety of practice settings, only California pharmacy residents were surveyed. This was meant to narrow the variability in residency stipends and scope of practice, but the results may be less applicable nationwide. We did not assess the number of residents that responded from any given program, so residents who exhibit similar changes in burnout due to being in the same program may have disproportionately impacted the results. Additionally, residents with difficult-to-manage workloads may have been too burned out to complete the survey despite possible compensation. Total resident staffing hours in each weekend shift, responsibilities on shift or types of shifts staffed, frequency of stretches without weekend days off, or shifts with disruptive hours may also vary between programs even if weekend staffing requirements are numerically the same. Furthermore, the total hours spent by residents working on residency-related activities during the weekday or non-clinical duties (e.g., projects) on the weekends was not captured, which could also contribute to burnout. These other sources of workload may also explain the lack of observed differences in those with no weekend staffing requirement. These additional factors were not captured in this pilot study as to alleviate response burden on each survey. Similarly, moonlighting, where residents voluntarily work extra shifts for additional pay, was not accounted for in this study due to additional socioeconomic factors that could contribute to decisions to moonlight. This is supported by the recent literature establishing links between socioeconomic status and burnout in postgraduate medical trainees in the United Kingdom [34]. We also did not account for the presence of wellness support programs or impact of compensatory days (i.e., getting the day off after a staffing shift) due to their high variability in implementation between residency programs. Future studies with narrow focus may benefit from capturing these characteristics and expanding the geographical scope of respondents.

Although no significant differences in burnout were found in this limited sample among those who did not have to staff weekends, it should be noted that there were demographic differences in certain key residency characteristics. Particularly, respondents who were not required to staff weekends were proportionally enrolled in PGY1 Ambulatory Care programs compared to PGY1 Acute Care programs. Those not required to staff weekends also self-identified in government and community health system residency programs with lower expected residency stipends. Since residency applicants may select programs based on above characteristics, future studies should consider performing separate analyses that account for each of these factors’ contributions to burnout. These results also suggest that certain types of programs and health systems are associated with less or no weekend staffing (e.g., Ambulatory Care residencies or Veterans Affairs’ governmental health systems); therefore, future studies examining the qualitative properties of the duties in these settings may help distinguish the contributions of weekend staffing to burnout. It is also possible that respondents who selected residencies for certain characteristics already have a higher magnitude of burnout, so future studies should consider elucidating what residency characteristics are important to applicants based on the presence of higher or lower burnout scores.

While unique stressors at two timepoints of the residency year may have been captured when burnout may have temporarily been higher, the cumulative impact of weekend staffing on burnout over time may not have been fully assessed. Notably, a nationwide survey of pharmacy residents in the residency cycle approximately 3 years prior to the cohort in our study demonstrated increasing rates of burnout over the course of the year when measured at four timepoints [35]. This study measured prevalence of burnout using the Oldenburg Burnout Inventory (OBI) and used a defined cutoff point to define an individual as burned out. In combination with our findings, which measures burnout from a magnitude perspective as opposed to prevalence with a dichotomous cutoff, both studies suggest that burnout worsens among pharmacy residents as the residency year progresses. Future studies should consider comparing different burnout scales used in pharmacy trainees and assessing both changes in magnitude and prevalence.

Since our study utilized an optional self-report questionnaire, there may have been limitations in self-reporting. Residents, especially those who staff by frequency and not a total number of weekends, may have inaccurately reported their weekends staffed. In future studies, confirming number of weekends staffed with RPDs at the end of the year rather than through self-report of expected number of weekends may alleviate this issue. This may also allow for analysis of optional weekend staffing (i.e., moonlighting).

## 5. Conclusions

Based upon this longitudinal cohort of California pharmacy residents, differences in burnout score changes did not seem to be associated with the number of weekends required to staff. Additionally, higher EE scores seem to be associated with PGY2 residents compared to PGY1 residents at the midpoint of residency year. Higher burnout scores may be associated with those who become less likely to pursue BPS certification over time. However, these findings should be interpreted cautiously given the geographical limitations to California residents, the small sample size due to an overall low response rate relative to the number of resident positions, and lack of data collected on potential confounding determinants of residency program experiences. Future studies should consider additional geographical scope and qualitative and quantitative comparisons to account for variations among residency program staffing requirements and characteristics. Future studies should also examine how burnout affects residents’ long-term engagement with professional endeavors.

## Figures and Tables

**Table 1:** Baseline demographics and residency program characteristics of respondents.

Variable	No Weekend Staffing (*n* = 11)	Weekend Staffing Required (*n* = 54) *	Overall (*N* = 65) *
Age, mean years (±SD)	26.4 (±3.2)	26.8 (±1.9)	26.7 (±2.2)
Weekends staffed, mean (±SD)	N/A	13.7 (7.4)	N/A
Gender Identity (%)			
Female	7 (63.6)	44 (81.5)	51 (78.5)
Male	3 (27.3)	10 (18.5)	13 (20.0)
Other	1 (9.1)	0 (0.0)	1 (1.5)
Ethnicity (%)			
Asian	6 (54.5)	31 (57.4)	37 (56.1)
White	5 (45.4)	11 (20.4)	16 (24.2)
Other	0 (0.0)	12 (22.2)	13 (19.7)
Residency Program Type (%)			
PGY1 Acute Care Focus	1 (9.1)	28 (51.8)	29 (44.6)
PGY1 Ambulatory Care Focus	4 (36.4)	5 (9.3)	9 (13.8)
PGY1 (Other)	1 (9.1)	4 (7.4)	5 (7.7)
PGY2	5 (45.4)	17 (31.5)	22 (33.9)
Primary Practice Setting (%)			
Academic Hospital/Health System	2 (18.2)	35 (64.8)	37 (56.9)
Government Hospital/Health System	5 (45.4)	5 (9.2)	10 (15.4)
Community Hospital/Health System	4 (36.4)	13 (24.1)	17 (26.2)
Other	0 (0.0)	1 (1.9)	1 (1.5)
Expected Residency Stipend (%)			
Less than $50,000	4 (36.4)	8 (14.8)	12 (18.4)
$50,000–$60,000	6 (54.5)	19 (35.2)	25 (38.5)
More than $60,000	1 (9.1)	27 (50.0)	28 (43.1)
Staffing Compensation Model (%)			
Required to take compensation day	N/A	12 (21.8)	N/A
Optional compensation day	N/A	7 (12.7)	N/A
No compensation day	N/A	36 (65.5)	N/A

* One respondent who had a weekend staffing requirement completed the primary outcome measure but did not complete all demographic information in the survey, therefore the total cohort analyzed was from 66 respondents but demographic information is only available for 65 respondents. Between PGY1 and PGY2 subgroups, *t*-tests revealed no statistically significant differences in mean age, and no statistically significant differences in ethnicity or gender identity were detected by chi-square test. SD = Standard Deviation. PGY1 = Postgraduate Year 1, PGY2 = Postgraduate Year 2.

**Table 2:** Subgroup comparisons of MBI-HSS [MP] domain scores baseline and final survey timepoints.

Overall Combined Cohort (*N* = 66)			
MBI-HSS [MP] Domain	Baseline MBI-HSS [MP] Survey Score	Final MBI-HSS [MP] Survey Score	*p*-Value (Two-Sided)
Emotional Exhaustion, mean (±SD)	24.8 (10.2)	28.4 (11.5)	* 0.036
Depersonalization, mean (±SD)	6.48 (4.50)	6.98 (6.41)	0.59
Personal Accomplishment, mean (±SD)	36.4 (6.34)	37.1 (5.44)	0.41
No weekend staffing vs. any required weekend staffing (*N* = 66)			
Baseline MBI-HSS [MP] Survey Score	No weekend staffing (*n* = 11)	Any required weekend staffing (*n* = 55)	*p*-value (two-sided)
Emotional Exhaustion, mean (±SD)	22.2 (10.5)	25.4 (10.1)	0.35
Depersonalization, mean (±SD)	7.36 (4.70)	6.31 (4.48)	0.48
Personal Accomplishment, mean (±SD)	37.6 (5.97)	36.3 (5.43)	0.53
Final MBI-HSS [MP] Survey Score	No weekend staffing (*n* = 11)	Any required weekend staffing (*n* = 55)	*p*-value (two-sided)
Emotional Exhaustion, mean (±SD)	26.5 (9.45)	28.8 (11.9)	0.57
Depersonalization, mean (±SD)	5.82 (5.00)	7.22 (6.67)	0.51
Personal Accomplishment, mean (±SD) (lower scores indicate greater experienced burnout)	36.2 (6.39)	37.3 (5.27)	0.53
PGY1 vs. PGY2, if required to staff weekends (*n* = 54)			
Baseline MBI-HSS [MP] Survey Score	PGY1 (*n* = 37)	PGY2 (*n* = 17)	*p*-value (two-sided)
Emotional Exhaustion, mean (±SD)	25.7 (10.6)	24.9 (9.57)	0.79
Depersonalization, mean (±SD)	6.35 (4.73)	6.18 (4.16)	0.90
Personal Accomplishment, mean (±SD)	35.9 (7.07)	37.0 (5.06)	0.56
Final MBI-HSS [MP] Survey Score	PGY1 (*n* = 37)	PGY2 (*n* = 17)	*p*-value (two-sided)
Emotional Exhaustion, mean (±SD)	25.8 (12.0)	35.1 (0.70)	* 0.007
Depersonalization, mean (±SD)	6.57 (6.44)	8.88 (7.18)	0.24
Personal Accomplishment, mean (±SD) (lower scores indicate greater experienced burnout)	37.1 (5.46)	37.6 (5.12)	0.77

Data analyzed by two-sided *t*-test. Statistically significant *p* values (*p* < 0.05) are denoted by an * asterisk. Lower scores on the Personal Accomplishment subscale indicate greater experienced burnout. SD = Standard Deviation. MBI-HSS [MP] = Maslach Burnout Inventory for Medical Personnel. Lower scores on the Personal Accomplishment subscale indicate greater experienced burnout. PGY1 = Postgraduate Year 1, PGY2 = Postgraduate Year 2. Of note, one respondent did not demographic data therefore did not specify residency year, therefore PGY1 vs. PGY2 subgroup analysis is out of n = 54 instead of n = 55.

**Table 3:** Final MBI-HSS [MP] scores stratified by professional pursuits at final survey timepoint.

Plans to Pursue Postgraduate Training	More Likely (*n* = 11)	Less Likely (*n* = 12)	Unchanged (*n* = 41)	*p*-Value
Emotional Exhaustion, mean (±SD)	31.6 (11.0)	25.1 (13.1)	28.1 (11.3)	0.42
Depersonalization, mean (±SD)	8.45 (7.02)	6.67 (7.90)	6.68 (5.60)	0.72
Personal Accomplishment, mean (±SD)	35.1 (3.45)	37.8 (5.88)	37.4 (5.83)	0.42
Plans to Pursue BPS Certification	More Likely (*n* = 7)	Less Likely (*n* = 8)	Unchanged (*n* = 45)	*p*-Value
Emotional Exhaustion, mean (±SD)	32.6 (6.50)	36.8 (14.0)	26.5 (11.1)	* 0.039
Depersonalization, mean (±SD)	4.29 (4.79)	11.9 (10.0)	6.56 (5.58)	* 0.047
Personal Accomplishment, mean (±SD) (lower scores indicate greater experienced burnout)	36.6 (3.21)	32.3 (8.01)	37.8 (5.08)	* 0.031

Total *n* for sample is less than for total MBI-HSS [MP] sample results as some respondents did not complete responses or reported they already had BPS Certification. Statistically significant *p* values (*p* < 0.05) are denoted by an * asterisk. Lower scores on the Personal Accomplishment subscale indicate greater experienced burnout. SD = Standard Deviation. MBI-HSS [MP] = Maslach Burnout Inventory for Medical Personnel. BPS = Board of Pharmacy Specialty.

## Data Availability

The data presented in this study are available on request from the corresponding author due to the confidentiality of the responses of participants in this study and the datasets presented in this article are not readily available.

## Abbreviations

The following abbreviations are used in this manuscript:

JD-R	Job Demands–Resources
RPD	Residency Program Director
PGY1	Postgraduate Year 1
PGY2	Postgraduate Year 2
MBI-HSS [MP]	Maslach Burnout Inventory Human Services Survey for Medical Personnel
BPS	Board of Pharmacy Specialty
EE	Emotional Exhaustion
DP	Depersonalization
PA	Personal Accomplishment

## Appendix A

**Table A1:** Qualtrics^XM^ Survey Tool.

Question Number	Question Text	Answer Choices/Notes
1	E-mail address (You will be entered for gift card drawing regardless of participation)	Free response
2	I consent to participating in the study (your responses are confidential and will not be individually reported)	□ Yes □ No Ends survey if answer is no.
3	I am required to staff on the weekends.	□ Yes □ No □ Other (free response) Skips to question 7 if answer is no
4	Enter the TOTAL number of weekends you are required to staff for the 2023–2024 residency year. If your staffing requirement is not based on a total number of weekends, select N/A.	□ Number of weekends (free response) □ N/A Skips to question 6 if a number of weekends is entered
5	Describe how often you are required to staff on the weekend (i.e., every x number of weekends, only on certain rotations etc.)	Free response
6	If you are required to staff a weekend shift, indicate any compensated day that you receive during the week (i.e., a project day). Select N/A if you are not required to staff weekends.	□ Yes—we MUST take a compensated day □ Yes—but the compensated day is optional □ No, I do not get a compensated day □ N/A (I am not required to staff weekends) □ Other (free response)
7	MBI-HSS [MP] Tool	Tool not provided in this report due to copyright material.
8	Please rate your agreement to each statement regarding your role as a pharmacy resident.	Matrix question with the options: Strongly disagree, somewhat disagree, neither agree nor disagree, somewhat agree, or strongly agree for each line I feel accomplished as a pharmacy resident. I made the right choice by choosing to complete a pharmacy residency. I feel prepared for my future career due to opportunities available to me during residency. I have an appropriate level of independence as a pharmacy resident. I feel valued as a pharmacy resident.
9	Are you planning to complete further postgraduate training after residency?	□ Yes, planning to apply to PGY2 □ Yes, planning to pursue other postgraduate training □ No □ Unsure (please describe reason) (free response)
10	Are you currently planning to pursue any type of BPS Specialty Board Certification in the future? (e.g., BCPS, etc.)	□ Yes □ No □ Unsure (indicate why) (free response) □ I already have BPS Specialty Certification
11	Select the option that best describes your pharmacy residency program.	□ PGY1—Acute Care Focus □ PGY1—Ambulatory Care Focus □ PGY1—Community □ PGY1—Managed Care □ PGY2
12	Select the primary setting of your residency program.	□ Community Pharmacy/Retail □ Hospital/Health-System—Academic □ Hospital/Health-System—Government □ Hospital/Health-System—Community □ Managed Care/Pharmacy Benefit Management □ Nursing Home/Long Term Care □ Other (free response)
13	Enter your age.	Free response
14	Which of the following most closely describes your gender identity?	□ Female □ Male □ Transgender Woman □ Transgender Man □ Non-Binary or Gender-Fluid □ Agender or Don’t identify with any gender □ Prefer not to answer □ Other (free response)
15	Which of the following best describes your ethnicity?	□ American Indian or Alaska Native □ Asian □ Native Hawaiian or Other Pacific Islander □ Black or African American □ Hispanic or Latino □ White □ Prefer not to answer □ Other (free response)
16	Which of the following best describes your expected residency stipend?	□ Less than $40,000 per year □ $40,000–$50,000 per year □ $50,000–$60,000 per year □ $60,000–$70,000 per year □ Greater than $70,000 per year

Multiple choice questions are single answer unless otherwise stated in question.
